# The importance of model systems: Why we study a virus on the brink of global eradication

**DOI:** 10.1371/journal.ppat.1006330

**Published:** 2017-04-20

**Authors:** Julie K. Pfeiffer

**Affiliations:** Department of Microbiology, University of Texas Southwestern Medical Center, Dallas, Texas, United States of America; The Fox Chase Cancer Center, UNITED STATES

I wouldn’t be surprised to find my work on a politician-generated list of “ridiculous, wasteful” research projects: “Scientists examine how poop bacteria activate polio, an eradicated virus.” I have worked on poliovirus for 16 years. We work on other viruses too, but poliovirus has led to some of our most important discoveries. Many people only know polio as a paralytic disease that was eradicated from the United States decades ago. So why are we studying an eradicated virus?

Poliovirus is an incredible model system. It grows like a weed. We can make virus stocks containing 10^10^ infectious viruses without even trying very hard. For perspective, if these 10,000,000,000 viruses were dollar bills, the stack would be 68 miles high. Working with poliovirus is safe due to vaccination. We can make targeted mutations in the genome and generate mutant polioviruses within days. There are mouse strains that can be infected. Most importantly, poliovirus has been studied for over 100 years. We know a lot about poliovirus and we have great tools in our toolbox. If you’re going to tackle a tough problem, it helps to have a great toolbox. For other fields, the ideal toolbox may be fruit flies, worms, or yeast. Collectively, these model systems have illuminated biology and have led to major advancements in human health. What have we learned using poliovirus?

As a postdoctoral fellow in Karla Kirkegaard’s lab, I used poliovirus to show that RNA viruses benefit from a sloppy replication strategy. Many viruses, including Ebola, Zika, and influenza, have RNA as their genetic material. Replication of RNA by viral enzymes generates mutations, like a fast typist without spellchecker. Most of these mutations are damaging to the virus, but some can be beneficial. By isolating a mutant poliovirus that generated fewer mutations, I showed that a sloppy replication strategy benefits the virus by conferring adaptability during infection of animals. Marco Vignuzzi, then in Raul Andino’s lab, published similar results. Long story short, Marco and I began as competitors but are now great friends (see https://storify.com/HarmitMalik/on-one-of-the-enduring-friendships-in-virology). Marco’s lab has since shown that other viruses, including chikungunya, also rely on sloppy replication for optimal fitness. Several groups are exploring viruses with altered fidelity as vaccine candidates. In the end, our work with poliovirus was applicable to many viruses and may lead to new vaccine strategies.

Over the past several years, my lab has shown that intestinal bacteria promote infection with poliovirus and other gut viruses. When we used antibiotics to deplete bacteria from mice, poliovirus replication and disease was reduced. We found that poliovirus sticks to bacteria, which aids viral infection and transmission. Tatyana Golovkina’s lab showed similar effects for another model virus, mouse mammary tumor virus, and our papers were published together. Since then, several groups have shown that a variety of gut viruses rely on intestinal bacteria for infection. One of my favorite examples is Stephanie Karst’s work with human norovirus. Norovirus infections are common and miserable, with explosive vomiting and diarrhea. However, we know relatively little about human norovirus. In spite of 40 years of effort, human norovirus could not be cultured in the lab, which severely limited progress. The only method to study viral replication was to infect human volunteers! Human stool samples were filtered to remove bacteria to generate “virus” preparations. Stephanie is a friend and knew about our poliovirus results. She added bacteria back to her virus samples, and then the virus could replicate in cultured cells. The ability to grow human norovirus in the lab is a huge advance that could lead to new treatment or prevention strategies. And it all started in small labs with curiosity-driven studies using poliovirus and mouse mammary tumor virus.

The value of model systems and curiosity-driven basic science research is immense, but we often fail to convey this to the general public, to those in political office, and even to other scientists. Some scientists put a premium on clinical or translational relevance. Although a project need not have disease relevance to have high merit, basic science grant proposals can suffer a penalty from biased reviewers. When communicating their work, many scientists start with a description of the disease burden of their pathogen to highlight significance, rather than discussing why their biological question is interesting or important. It’s easy to understand why some nonscientists have trouble understanding the value of model systems when some scientists have the same problem. A diversified portfolio of research projects is needed—translational and basic science, model systems and disease models, and “big data” collaborative projects and investigator-initiated projects. But I hope that there will always be support for scientists that “geek out” over fruit flies, worms, yeast, or nearly eradicated viruses.

**Image 1:**
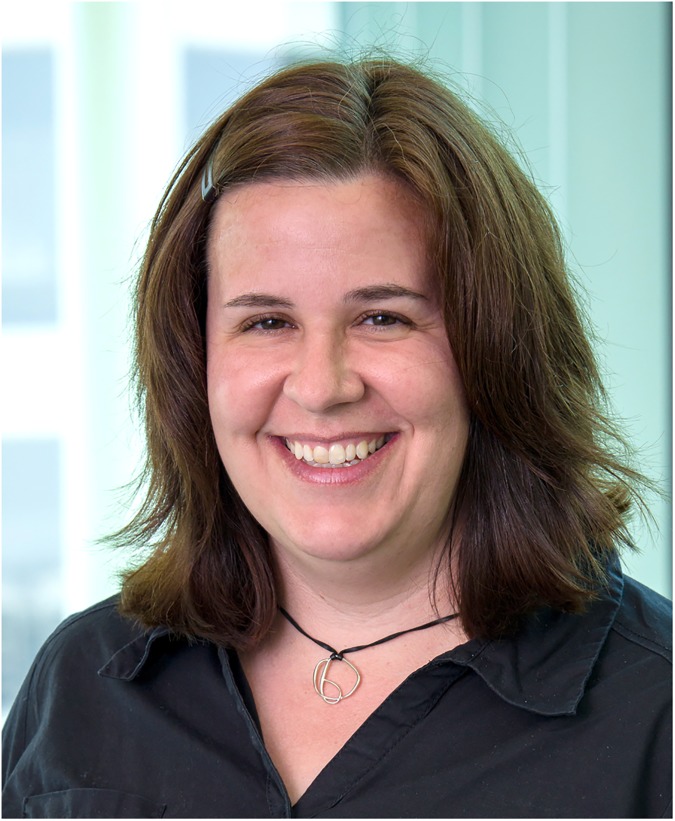
Julie Pfeiffer.

